# What Constitutes the High-Quality Soundscape in Human Habitats? Utilizing a Random Forest Model to Explore Soundscape and Its Geospatial Factors Behind

**DOI:** 10.3390/ijerph192113913

**Published:** 2022-10-26

**Authors:** Jingyi Wang, Chen Weng, Zhen Wang, Chunming Li, Tingting Wang

**Affiliations:** 1 Fujian Key Laboratory of Watershed Ecology, Key Lab of Urban Environment and Health, Institute of Urban Environment, Chinese Academy of Sciences, Xiamen 361021, China; 2 College of Resources and Environment, University of Chinese Academy of Sciences, Beijing 100049, China; 3 School of Statistics, Huaqiao University, Xiamen 361021, China

**Keywords:** soundscape, geospatial factors, machine learning, classification prediction, partial dependence analysis

## Abstract

Soundscape is the production of sounds and the acoustic environment, and it emphasizes peoples’ perceiving and experiencing process in the context. To this end, this paper focuses on the Pearl River Delta in China, and implements an empirical study based on the soundscape evaluation data from the Participatory Soundscape Sensing (PSS) system, and the geospatial data from multiple sources. The optimal variable set with 24 features are successfully used to establish a random forest model to predict the soundscape comfort of a new site (F1 = 0.61). Results show that the acoustic factors are most important to successfully classify soundscape comfort (averaged relative importance of 17.45), subsequently ranking by built environment elements (11.28), temporal factors (9.59), and demographic factors (9.14), while landscape index (8.60) and land cover type (7.71) seem to have unclear importance. Furthermore, the partial dependence analysis provides the answers about the appropriate threshold or category of various variables to quantitatively or qualitatively specify the necessary management and control metrics for maintaining soundscape quality. These findings suggest that mainstreaming the soundscape in the coupled natural–human systems and clarifying the mechanisms between soundscape perception and geospatial factors can be beneficial to create a high-quality soundscape in human habitats.

## 1. Introduction

The term “soundscape” was firstly proposed in the late 1960s by R. Murray Schafer, a Canadian musician. It was initially referred to as “The Music of Environment”, regarding the whole world as a macro music piece and advocating the combination of noise reduction and positive soundscape creation [1]. Nonetheless, studies are confined to conceptions aiming to arouse people’s awareness at this stage. In practice, the implementation of soundscape planning was originally only embodied in noise control, the particular negative aspect of the soundscape. The Environmental Noise Directive (END) raised by European Union in 2002 takes the most large-scale action. It established rules of measurement and management of environmental noise in the form of legislation, requiring the Member States to prepare and publish noise maps and noise management action plans every five years [2]. In 2014, making use of the available results of the second round of noise mapping in the framework of END (version August 2013), they assessed the health implications of environmental noise, including annoyance, sleep disturbance, reading impairment, hypertension, coronary heart disease and stroke [3]. These series of actions eventually caused great concern from the communities of academy and practice.

As researchers realized that noise maps do not include preferred sounds, such as the wind in trees, purling water, or birdsong perceived as enjoyable, their attention has gradually moved to the positive facets of the soundscape [4]. The U.S. National Park Service (NPS) recognizes soundscape as a resource while considering “natural quiet” as the ideal state of soundscape protection and management in the national park [5]. Despite the inspiring work by NPS, the realm of positive soundscape planning practice is still limited in the natural areas. Yet, in the areas of human habitats, such as urban and its peripheral regions, the overall soundscape planning, including both negative and positive ones, has not fallen into regulation or practice.

However, soundscape can largely influence human physical and mental health, thus relative issues in areas with intensive human activities should be extensively taken into account. The World Health Organization (WHO) has implemented Environmental Noise Guidelines for the European region. The findings show that noise is the second largest environmental cause of health problems, just after the impact of air quality (particulate matter). Various researchers endeavor to unravel the correlation between noise and several health outcomes—cardiovascular and metabolic effects, annoyance, effects on sleep, cognitive impairment, hearing impairment and tinnitus, adverse birth outcomes and quality of life, mental health and well-being—and different degrees of significance were demonstrated [6,7,8,9]. Similar effects have also been verified regarding positive soundscape, normally in the form of natural sounds. Buxton et al. [10] conducted a systematic literature review and meta-analysis. The results indicated that water sounds had the most significant effect on health and positive affective outcomes, while bird sounds had the most significant impact on alleviating stress and annoyance based on Attention Restoration Theory and Stress Recover Theory. They asserted that natural sounds as a sort of natural services’ provider.

According to these pivotal health effects, soundscape planning in human habitats appears to be an urgent need, despite facing great challenges due to the complexity of human–nature interaction. In the view of theory development, the acoustic researchers focus on the objective attribute of the sound environment and the reduction of noise. In contrast, the soundscape researchers are concerned with people’s subjective experiences, especially after the precise definition of soundscape emphasizing perception was confirmed by International Standardization Organization (ISO) in 2014 [11]. Simultaneously, Pijanowski et al. [12] initiated the discipline of soundscape ecology, putting soundscape into the coupled natural–human systems to reveal its spatial and temporal patterns, which systematically integrates the objective and subjective attributes of the soundscape. This tendency implies that the soundscape story is far beyond the acoustic elements, but also the surrounding context; in the case of the human habitat—the coupled natural–human systems.

In this sense, it is critical to deconstruct the complexity of human habitats. Many case studies have analyzed the correlation between soundscape and diverse spatial influence factors. The investigated influence factors broadly include different geospatial elements; to name just a few, Brambilla et al. [13] carried out an experiment in urban squares to assess the soundscape on the foundation of two environmental features, that is “chaotic/calm” and “open/enclosed”, which are obtained from L_Aeq_, loudness, roughness, sharpness and the geometry of the square (S/H ratio) through the principal component analysis (PCA), and the result shows a good correlation between physical parameters and subjective ratings. Liu et al. [14] verified the close relationships between soundscape perception and landscape composition indicators including the density of construction (CD), main roads (RD), and vegetation greenness (NDVI), as well as landscape configuration indicators including landscape shape index (LSI) and distance to constructions (DTC) and main roads (DTR), among which NDVI and LSI are two most important. Hong et al. [15] explored the relationship between spatiotemporal characteristics of soundscape and acoustic indicators, including L_Aeq_, L_Ceq-Aeq_, L_10–90_, and sharpness, as well as urban morphological indicators relating to buildings, roads, open public spaces, and water feature components in the multifunctional urban areas. Results indicate that the application of a combination of these two factors could be a promising approach for developing soundscape prediction models. Zhao et al. [16] constructed a structural equation model for the influence of urban contextual factors (namely, urban management, natural and urban conditions, and the physical environment) on soundscape evaluation (including pleasantness and eventfulness) based on the positive relationship being found. However, the previous researches are restricted to a single or small-scale study area, and the limited influence factors impede the application and promotion of conclusions. What is now necessary is the further development and integration of this knowledge in a larger-scale context, as well as a more comprehensive potential data set of explanatory variables.

This paper combines the ISO definition of soundscape and the conceptual framework of soundscape ecology, mainly regarding subjective soundscape quality as the dependent variable and objective geospatial factors as independent variables. Hereby, we can integrate soundscape into a systematic coupled natural–human system and draw a clear-cut picture of it. The main object is to strive for an appropriate approach to predict soundscape quality which can handle a large set of explanatory variables and sample data generated from urban environment. To this end, random forest as a potential effective machine learning method is utilized to build the classification model of soundscape perception, identify the importance of variables, and uncover the nonlinearities influence of those variables on the soundscape perception, as well as the interaction mechanism among variables. Our research will contribute to providing an exploratory methodology of the random forest model committing to soundscape quality classification and prediction, as well as the results with practical significance to reveal the hidden variables behind high-quality soundscape based on our empirical study.

## 2. Materials and Methods

### 2.1. The Participatory Soundscape Sensing System and the Soundscape Quality Proxy

The PSS system [17] is a worldwide soundscape investigation and evaluation project initially initiated in 2011 and fully upgraded in 2016. The concept of Participatory Sensing (PS) is the process whereby individuals and communities use the increasingly convenient mobile phones and cloud services to collect, analyze, and contribute sensory information for use in discovery [18]. For this, PSS encourages citizens worldwide to participate in standardized data collection and calculation tasks with the aid of SPL Meter and mobile networks. The data is collected in the means of automatic acquisition from mobile phones and questionnaire user surveys, including Sound Pressure Level (SPL) calculation, location, spatial attribution, subjective evaluation, individual characteristics, and other supplementary elements. All the measurements can be stored on mobile phones or shared with the PSS server.

Specifically, using a 5-point score scale, the questionnaire survey collects the subjective evaluation in terms of sound level, sound comfort level, sound harmonious level, and identified sound source. Given the comprehensibility of the perception indices, the public has a common understanding about the sound comfort levels and thus higher data reliability is observed, while sound harmonious level, which was originally designed to evaluate the harmony between visual landscape and auditory soundscape, seems to cause misunderstanding. For instance, the subjects may consider the industrial noise in an industrial area as disharmonious, which is actually harmonious in such a context. Therefore, we take sound comfort level to indicate the soundscape perception information and as the proxy of soundscape quality, with the degree from very uncomfortable to very comfortable, ranking the score from −2 to 2.

**Figure 1:**
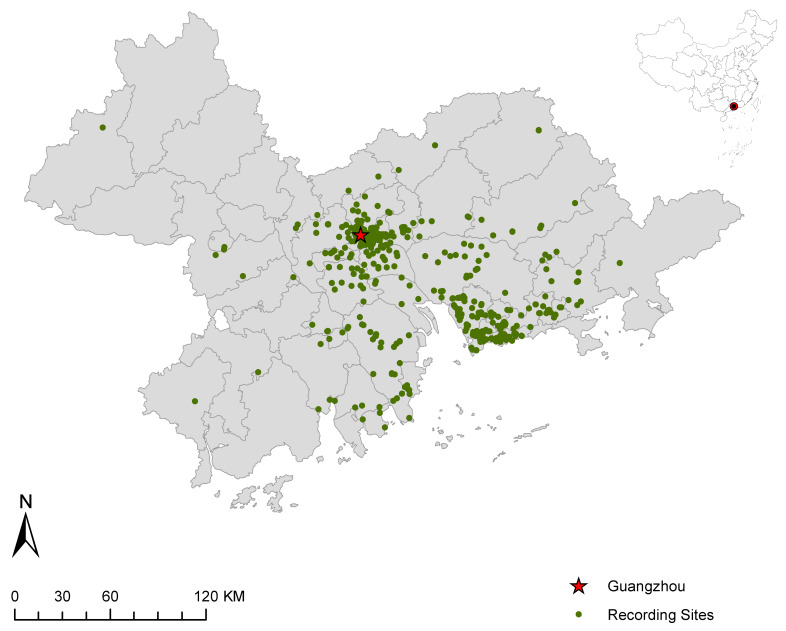
Distribution of recording sites.

Given the data availability of all influence factors, we take the Pearl River Delta of China as the research area in our case study. The Pearl River Delta, named as Southern Gate of China, covers an area of 56 thousand km^2^, and houses about 78.01 million people (the 7th national population census), occupying one third of and 61.9% of the total value of Guangdong province, respectively. Together with Hong Kong and Macao, the Pearl River Delta with nine main cities can expand to the Greater Bay Area, which is one of the top four bay areas in the world, as well as one of the largest mega-urban agglomerations worldwide. After removing invalid data with null values or valueless inquiries, such as the records with incompletely filled out inquiries, incorrectly acquired acoustic measurements, and duplicate records at the same location, 614 complete records from the PSS project remain (see Figure 1).

### 2.2. Multi-Source Geospatial Data and Explanatory Variables

Geospatial data with location coordinates in a spatial referencing system describes objects and things in relation to geographic space [19]. Longitude and latitude provide the basic information of geospatial position. Several anthropogenic elements in the built environment including roads, buildings, POI (points of interest), and land use type can reflect urban development intensity and human disturbance from the perspective of geospatial function, while the land cover and its configuration characteristics measured by the landscape index can depict the landscape surface from the perspective of geospatial configuration. Finally, nighttime light value can provide a comprehensive understanding of social-economic development on the different geospatial scales.

We adopted multiple data sources to maximize data availability including Global NPP-VIIRS-like Nighttime Light Data, GlobeLand 30, and major map service providers in China including Amap and Baidu Map. Global NPP-VIIRS-like Nighttime Light Data is a new flourishing data set with a spatial resolution of 15 arcsec (~500 m) which has been verified to show a similar spatial pattern as the composited NPP-VIIRS NTL data. It is built through a new cross-sensor calibration from DMSP-OLS NTL data (2000–2012) and a composition of monthly NPP-VIIRS NTL data (2013–2018). It can be better used to evaluate and analyze the dynamics of demographic and socioeconomic characteristics during urbanization [20]. GlobeLand 30, the 30 m resolution global land cover data product, was developed by China. It adopts the WGS-84 coordinate system and includes ten land cover classes: cultivated land, forest, grassland, shrub land, wetland, water bodies, tundra, artificial surface, bare land, perennial snow, and ice. The total accuracy of land cover classification is 85.72%, and the Kappa coefficient is 0.82 [21].

We finally organize 36 potential explanatory variables based on the principles to better reflect the capacity of the biological habitat, the intensity of human activities, the spatial configuration of an urban area, and the overall vitality of the social economy. The corresponding evaluation indices and their formulas for these variables are listed in Table 1. In addition, we defined an analytical unit (AU), which is a 1 km diameter circular buffer with the recording site as the center (0.7854 km² exactly), to calculate the indices effectively.

(1) Road density and relative distance

Road, accompanied by traffic noise, is the primary source of anthropogenic noise. Road density and distance between objective and road are the classical indices to estimate the traffic intensity and influence. Nonetheless, they neglect the distinction of traffic capacity among different road hierarchies. However, research has shown that road traffic flow can cause different soundscapes [22]. Thus, we further considered the road class as the weight and separately discussed the impact of major roads and express roads on the soundscape. The attribution of road class can be obtained from OSM with 27 classes in total, which are reclassified into four classes for more convenient analysis, including major road, minor road, express road, and other roads. According to the different hierarchies of road classes, the weighted scores of the express roads, major roads, minor roads, and other roads are given as 0.4, 0.3, 0.2, and 0.1, respectively.

(2) Three-dimensional building structure

Buildings are the major artificial constructions in the human habitat, and their existence can impact population density and human activity intensity. Human constructions could further result in a fragmented and diverse landscape, both of which can significantly affect soundscape patterns and experience. Moreover, three-dimensional elements such as roof types, ground properties, wind flow, and turbulence also affect sound propagation [23]. Given the data availability, we used the floor number of the building divided by floor area to characterize its three-dimensional structure which affects the sound transmission from the external environment and finally forms a specific local soundscape pattern.

(3) Type of land use and functional entropy of POI

The anthropogenic portion of soundscape is increasingly considered a kind of disturbance, resulting in the acoustic signal alteration in birds and amphibians and changes in reproductive behavior [24]. Researchers have discussed the correlation between soundscape and characteristics of land use such as the landscape development intensity indices and have drawn a statistically significant result [25]. In addition, spaces with plentiful POI and high-intensity human activities can intrinsically emanate soundscape with a larger proportion of anthrophony. We manually identified the land use type of the recording site and that of the context in the AU based on Google satellite remote sensing maps to point out how humans develop land and what functions the spaces provide. Meanwhile, based on the POI data, we constructed an entropy index to describe the disorder of land use and spatial function.

(4) Nighttime light value

Nighttime light remote sensing data can detect the artificial lights from cities, towns, and industrial areas through the sensors’ recordings [26] and have demonstrated good performance in representing human socioeconomic activity and human development [27,28]. Therefore, we utilized the NPP-VIIRS-like NTL data (version 2016-version 2020) as a comprehensive reference to reflect the overall development level in the built environment.

(5) Proportion of different land cover

Forests, trees, shrubs, tall grass prairie or other grasslands, and other vegetation types are proved to be beneficial for noise attenuation and useful as sound barriers for urban planning [29]. Researchers have evaluated the effect of water sounds on soundscape in urban areas, and the results found that water sounds from water features like fountains, streams, water sculptures, or waterfalls with relatively greater energy in low-frequency ranges were effective for masking noise caused by road traffic [30]. In addition, the distribution of bird species is significantly related to the proportion of different land cover types [31], indicating that the green and blue spaces can provide habitats and activity places for organisms and humans, respectively, which can produce biophony, geophony, and anthrophony of the soundscape. Thus, we adopted the data from GlobeLand 30 (version 2020) to explore the hidden soundscape on the base map of different land cover types, including artificial surfaces, cultivated land, grassland, forest, shrub land, and water bodies. The types of bare land, wetlands, tundra, as well as glaciers and perennial snow were left out due to especially small sample sizes.

(6) Landscape index

Many studies have suggested a close relationship between soundscape and landscape perception in terms of aural–visual interaction [32,33,34,35]. On the other hand, research has also proved that landscape can affect the perception of certain sounds to a different extent, mainly through landscape features like normalized difference vegetation index (NDVI), landscape shape index (LSI), patch density (PD) and Shannon diversity index (SHDI) [14]. Here, we selected several classical landscape indexes to indicate landscape features from three major aspects: landscape fragmentation or connectivity (including PD, ED, MPS, LPI, CONT), landscape structure complexity (LSI), and landscape diversity or richness (including SHDI and PR) [36].

In addition to the above-mentioned geospatial factors, we also listed the selected data types collected by the PSS system as the potential explanatory variables. Acoustic factors, including a subjective evaluation of sound source and objective measurement of SPL, are selected to discuss the relationship between acoustics emphasizing the physical aspect and soundscape emphasizing the perceptive aspect. Demographic factors, including age and gender, are used to explore whether discrimination against soundscape experience exists among the different groups. Finally, we divided the recordings from the PSS system according to diurnal and seasonal periods to better describe the subtle difference in soundscape experience based on a temporal scale.

### 2.3. Method of Random Forest Model

The development of machine learning promotes the maturity of techniques in terms of nominal predictive power, interpretability, tunable parameters, robustness, and capacity to deal with mixed data types and potentially irrelevant inputs. Random forest (RF), a machine learning method based on an ensemble of many individual decision trees, performs well in handling a very large number of explanatory variables [37]. The RF algorithm applies the idea of bootstrap aggregating (Bagging) methods to the classification and regression tree (CART) algorithm by creating new training sets with random sampling (bootstrap sample). Each classification tree of RF is generated by resampling the original records with replacement, in which process one third of the data will be left out to naturally form a comparison data set serving as cross-validation. This unused subset of bootstrap sample is called out-of-bag data (OOB); as an unbiased estimation of prediction error, the OOB error is used for model accuracy evaluation. Compared to multiple linear regression, spatial auto-correlation, geographically weighted regression (GWR), Bayesian regression, and artificial neural networks (ANN), random forests have a lower computation without losing prediction accuracy, a higher tolerance to outliers and noise, a faster-operating rate to deal with high-dimensional data and can effectively avoid overfitting. Moreover, there are no requirements for the prior information about the form of relationship and interaction among variables [38].

Mennitt et al. [39] presented a geospatial based on a random forest model that predicts acoustical measurements using various geospatial features across the contiguous United States, and the preliminary results suggested that tree-based methods, including random forest, had more promise than linear models, generalized additive models and support vector machines.

According to the previous study, we utilize the randomForest and rfPermute algorithm in R to establish the potential classification model of the aforementioned soundscape data and the multi-source geospatial data and provide the evaluation results of the model performance.

The randomForest algorithm implements Breiman’s forest algorithm for classification and regression. The rfPermute packages the randomForest algorithm while it can further provide estimated significance of importance metrics for a random forest model by permuting the response variable. By setting the parameter of *num.rep*, the response variable is then permuted according to the set times, with a new random forest model built for each permutation step. We combined both algorithms to acquire the necessary information. The model construction process included three stages: tuning the optimized model parameters, assessing the model accuracy, and ordering the variables’ importance. It is critical to note that the first two steps should be a circular and repeating process.

(1) Tuning the model parameters and constructing the rudimentary model

Several parameters regulate the structure of a random forest model [40]: the number of variables randomly sampled as candidates at each split (*mtry*), the number of trees to grow (*ntree*), the sample size presented to each tree (*sampsize*), and the minimum size of terminal nodes (*nodesize*). To solve the data imbalance, we merged the comfort score into three levels: uncomfortable (scores of “−2” and “−1”), moderate (score of “0”), and comfortable (scores of “1” and “2”), which has a sample size of 313, 232, and 69, respectively.

(2) Assessing the accuracy of the rudimentary model and re-tuning the model parameters until acquiring the optimal model

However, due to the still-existing imbalance, the estimation of OOB error can be misleading, which will manifest as an overall lower OOB error with a low recall rate of a certain level. We thus defined an average F1 score based on the confusion matrix calculation, which can take an integrated consideration of both the overall OOB error and recall rate of each three levels, and finally used to assist with the parameter setting and to evaluate the model performance. Moreover, by artificially making class priors equal and setting a unified *sampsize*, the random forest model will repeatedly draw the same sample size from all three strata, which is the third strategy to solve data imbalance [41]. The next step is to tune the four major parameters according to the performance of F1 scores.

(3) Ordering the variables’ importance

The relative importance and the significance of the explanatory variables can be acquired by rfPermute algorithm. There are two evaluation indicators: Mean Decrease Accuracy (MDA) and Mean Decrease Gini. The mean decrease accuracy expresses how much accuracy the model losses by excluding each variable. The more the accuracy suffers, the more important the variable is for the successful classification. The mean decrease in the Gini coefficient is a measure of how each variable contributes to the homogeneity of the nodes and leaves in the resulting random forest [42].

### 2.4. Method of Partial Dependence Analysis

While determining predictor importance is a crucial task for supervised learning problems, ranking variables can only tell part of the story, and once a subset of features is identified based on relative importance, it is necessary to assess the relationship between them and the response. In the realm of machine learning, particularly for black box models like random forests and support vector machines, the Partial Dependence Plots (PDPs) is an effective way to visualize the relationship between a subset of the features and the response. Specifically, it shows how the average response varies with a given predictor while the values of all other predictors are fixed at their base levels [43,44]. For a prediction model with a feature set *X*, we consider an interest set $Z_{s}\subset X$ and the complement subset $Z_{c}=X\backslash Z_{s}$. For each x∈X, $\hat{y}\left(x\right)$ is the response of the model, which is the random forest model in our case. Given any certain permuted value of $z_{s}\in Z_{s}$, the partial dependence function $\bar{\hat{y}}\left(z_{s}\right)$ is the average response of the model over all of the available training data for permuted values of $Z_{c}$. The $\bar{\hat{y}}\left(z_{s}\right)$ can be computed through formula (1).

(1) $$\bar{\hat{y}}\left(z_{s}\right)=\frac{1}{\left|Z_{c}\right|}\sum_{z_{c}\in Z_{c}}\hat{y\;}\left(z_{s},z_{c}\right).z_{s}\in Z_{s}.$$

In this study, we sought to tackle a three-categories classification problem. For each category, we calculate the partial dependence function ${\bar{\hat{y}}}_{i}\left(z_{s}\right),\;i=1,2,3$. The influence of the predictor $Z_{s}$ can be quantified by specifying a sequence of $z_{s}\in Z_{s}$ and calculating ${\bar{\hat{y}}}_{i}\left(z_{s}\right)$ for each, which is reflected as the color in the plots of the partial dependence function. In other words, the color of the PDP plots shows how the average response varies with a given predictor $Z_{s}$ while the values of all other predictors, $Z_{c}$, are fixed at their base levels. To conduct a marginal effect of single predictor, $Z_{s}$ consists of one certain variable, while in the case of joint effect and interactions among predictors the subset $Z_{s}$ is allowed to include multiple variables. It should be further noted that none of the causal relationship can be claimed.

## 3. Results

### 3.1. Model Construction and Importance Ranking of Variables

According to the above procedures of model construction, we acquired the optimal random forest model by repeatedly combing model accuracy assessing and model parameter tuning. Result shows that the random forest model is optimized when *mtry* is 14, *ntree* is 800, and *sampsize* is 63, while *nodesize* seems to have a marginal impact on F1 which is finally set as the default value. The F1 score of the final random forest model is 0.64.

**Figure 2:**
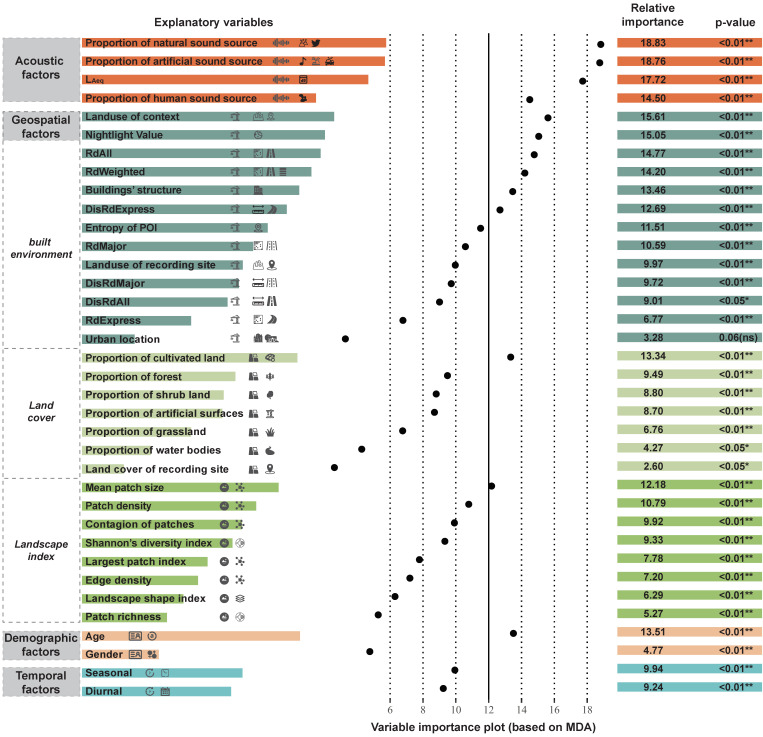
The relative importance and their corresponding significance of the explanatory variables for the overall soundscape comfort (**: *p*-value ≤ 0.01, *: *p*-value ≤ 0.05, ns: *p* > 0.05).

Then, we estimated the relative importance and the significance of 36 explanatory variables using the rfPermute algorithm, and selected the Mean Decrease Accuracy as the relative importance indicator. Results show that, excluding the variable of urban location, all the explanatory variables appear to be significantly important for successfully predicting soundscape comfort. As shown in Figure 2, the sequence of variables is ordered by their categories and then the relative importance, among which the importance value of acoustic factors is the highest (average of 17.45), then the built environment (average of 11.28), temporal factors (average of 9.59), demographic factors (average of 9.14), landscape index (average of 8.60), and land cover (average of 7.71) in sequence.

A separate subset of variables’ importance is listed in Table 2 for each level of comfort score. As the most important variable of the overall comfort score, acoustic factors have different impacts on the three levels. The proportion of natural sound sources is essential to successful identification of the comfortable soundscape, while in comparison the proportion of artificial sound sources can effectively judge whether the soundscape is uncomfortable. This indicates that a soundscape with wind, water, rain, insects, or birds can bring an enjoyable perception, while a soundscape with traffic, construction, or machine tends to cause a terrible soundscape impression. Across the top ten important variables, nighttime light value is the only geospatial factor that maintains high relative importance among all three comfort levels, yet other subclasses of geospatial factors differentiate slightly, which implies that nighttime light may be a valuable composite indicator to comprehensively reflect the general social-economic condition of the specific context.

### 3.2. Optimal Variable Sets and the Optimized Random Forest Model

Although the method of random forests is relatively insensitive to superfluous variables, unnecessary variables are not beneficial to enhancing the predictive power of models, and redundant variables are a burden for data accessibility and index calculation. In addition, a simple and clear subset of variables can be more feasible to interpret and more convenient to take into the application.

**Figure 3:**
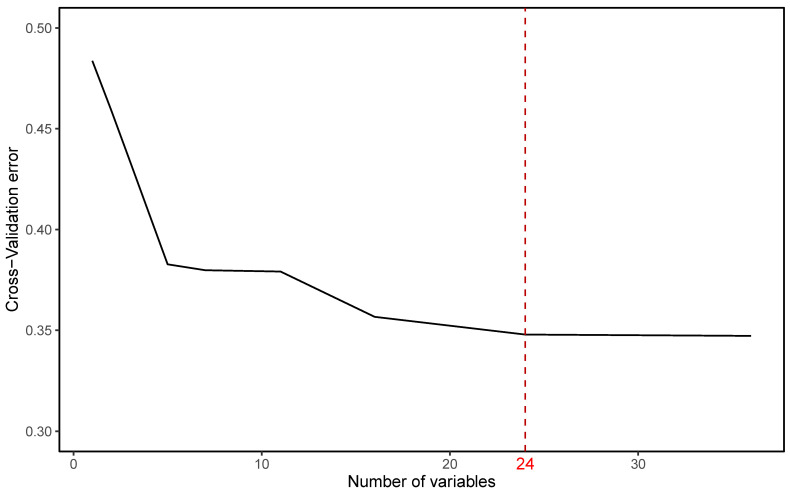
Determining the optimal variable set for the soundscape comfortable model based on CV error.

To determine the specific number of variables for an optimal model with great predictive ability, a ten-fold Cross-Validation (CV) was implemented, and the result shows that the CV error tends to maintain stability after the number of variables reaches twenty-four (see Figure 3).

Therefore, we selected the top 24 variables according to the relative importance measured by mean decrease accuracy as the optimal variable set, which contains eleven built environment factors (including land use of context, NLValue, RdAll, RdWeighted, BldStructure, DisRdExpress, POIEntropy, RdMajor, land use of the recording site, DisRdMajor, and DisRdAll), four landscape indices (including AREA_MN, PD, CONTAG, and SHDI), two land cover factors (including the proportion of cultivated land and that of forest), all of the four acoustic factors, all of the two temporal factors, and one demographic factor (age).

The model parameters are tuned again through the aforementioned process, and the final parameters are 8 of *mtry*, 800 of *ntree*, and 60 of *sampsize*. The new random forest model achieved the F1 score of 0.61. Despite the slight decrease, the elimination of twelve insignificant and unnecessary variables out of the original subset of thirty-six can largely simplify the prediction of soundscape comfort at a new site.

### 3.3. The Influence Mechanism between Explanatory Variables and the Soundscape

To explore the influence mechanism between explanatory variables and the soundscape, we utilize a general *R* package, *PDP,* to conveniently implement partial dependence analysis, and help understand the outcomes of the random forests model. Hereby we selected numbers of variables which are comprehensible for urban planning, and conducted the partial dependence analysis of the response on a single predictor, as well as the joint effect of several pairs of variables.

(1) The marginal effect of a single predictor on soundscape comfort evaluation

**Figure A1:**
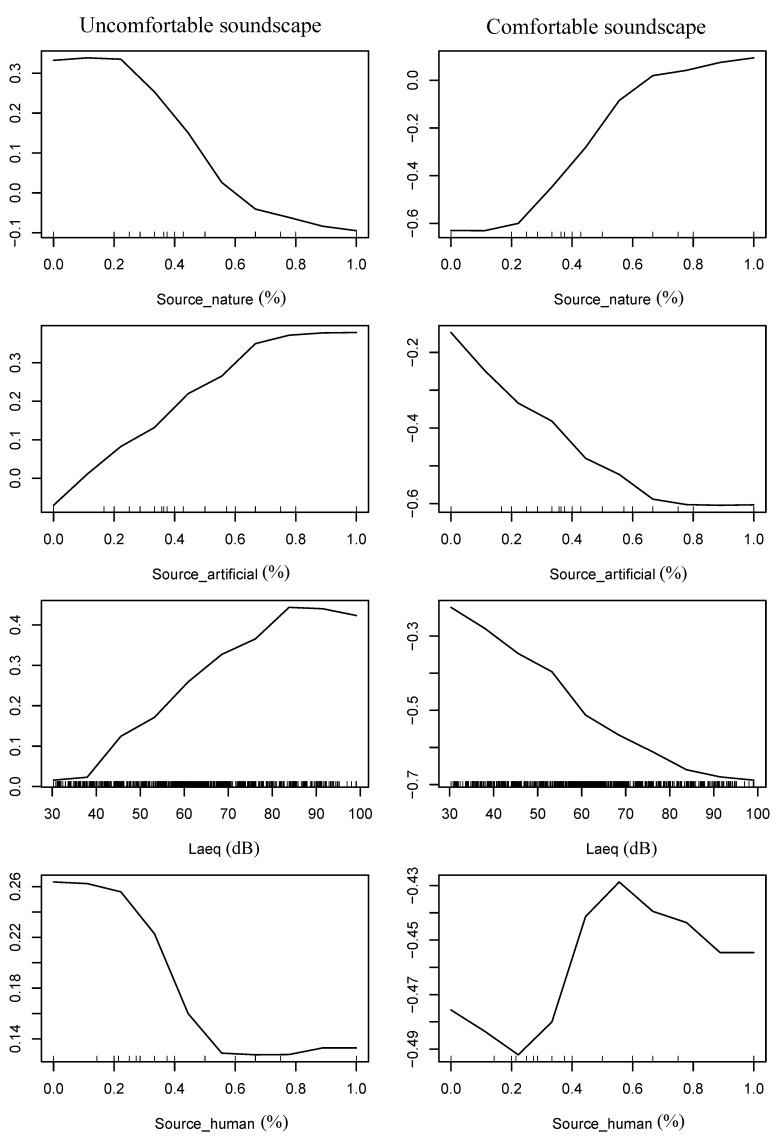
Partial dependence plot to understand the marginal effect of a single predictor on model prediction outcomes.

Acoustic factors most directly influence soundscape perception, mainly through the way of the content and the intensity of the sound. As shown in Figure A1 (in Appendix A), after the proportion of the natural sound source reaches 0.6, it will be more likely to lead to a comfortable perception while the probability to be recognized as an uncomfortable soundscape stably remains the lowest. Both the influence tendency of the proportion of artificial sound sources and the A-weighted equivalent continuous sound level (L_Aeq_) on discriminating between comfortable and uncomfortable soundscapes take a symmetrical form, which shows that a higher proportion of artificial sound or a higher L_Aeq_ will gradually cause a lower probability of comfortable perception and a higher probability of uncomfortable perception. In contrast with these three measurements, the proportion of human sound sources does not appear a prominent and consistent preference for soundscape perception. However, it is reasonable to conclude that in the case of human sound taking up the percentage of 0.2 to 0.6 the probability of comfortable soundscape will increase and that of uncomfortable will decrease, which indicates that it is more appropriate to maintain a moderate proportion of human sound.

**Figure A2:**
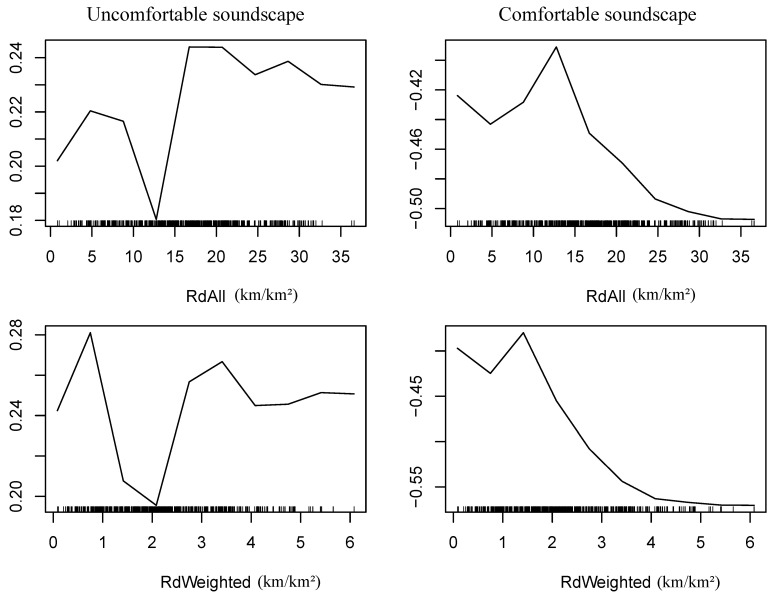
Partial dependence plot to understand the marginal effect of a single predictor on model prediction outcomes.

Geospatial factors have a more complex influence on soundscape perception, incorporating aspects of indirect ways and direct ways. These factors primarily provide the context of soundscape, which largely determine the underlying and overall impression of soundscape perception. Meanwhile, it defines the sound contents, that is, sound sources and their intricacy level, through the way of sound generation and sound propagation. Furthermore, it can directly change the sound intensity, such as in the case of the distance to the roads. In each AU, if the road density is higher than 15 km/km^2^, or the weighted road density with consideration of different road hierarchies is higher than 2 km/km^2^, the soundscape will tend to be increasingly uncomfortable (see Figure A2). The difference value of 13 km/km² here implies that people’s soundscape perception may be less tolerant to the existence of roads with greater weight such as the express roads and the major roads.

**Figure A3:**
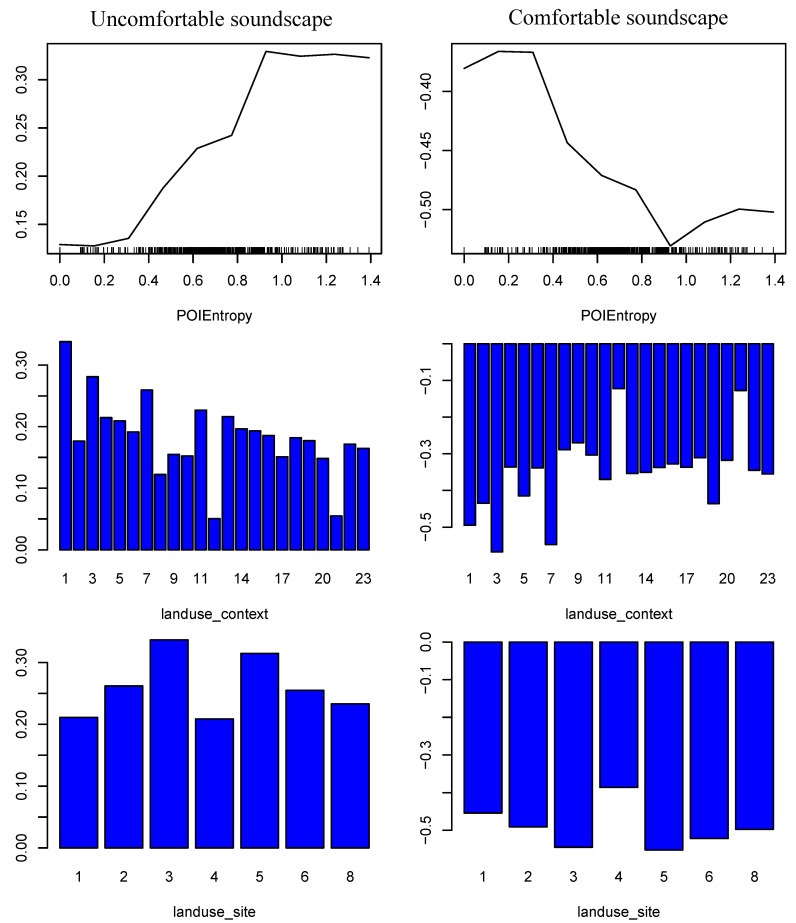
Partial dependence plot to understand the marginal effect of a single predictor on model prediction outcomes.

Both the entropy of POI and the type of land use can reflect the land context, and results show that to create a comfortable soundscape, POI entropy in each AU must stay below the level of 0.4. The land use type of a double functional area with residential combined with green space and the scenic spot is preferred for a comfortable soundscape, or it is also enjoyable if the person is located in a public administration and service (mostly college in our data set). In contrast, locations in an industrial area or a transportation area will significantly end up with uncomfortable soundscapes (see Figure A3).

**Figure A4:**
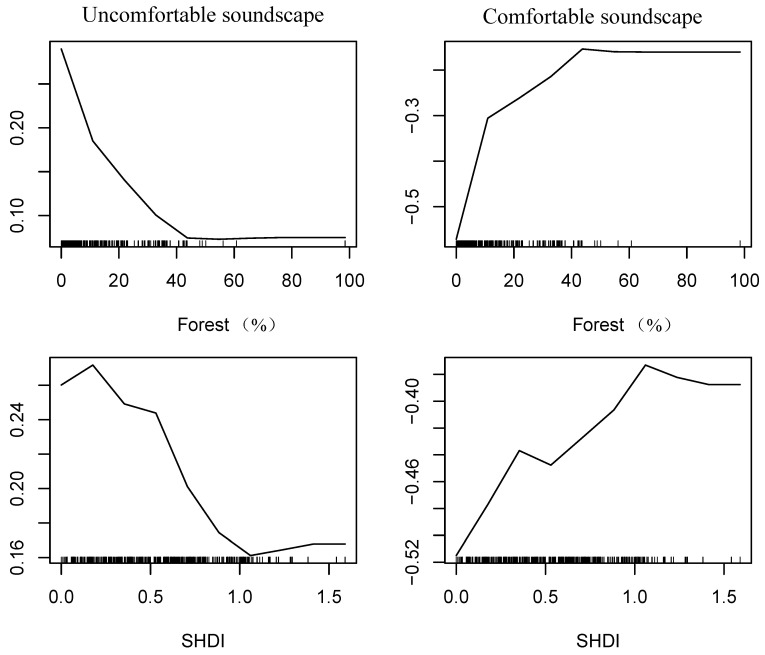
Partial dependence plot to understand the marginal effect of a single predictor on model prediction outcomes.

**Figure A5:**
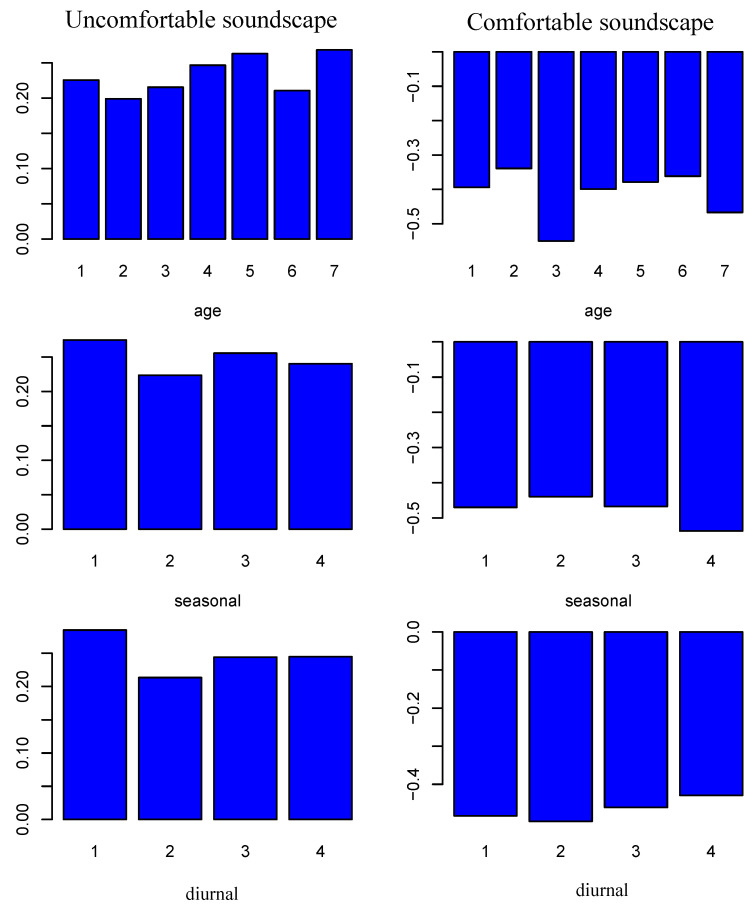
Partial dependence plot to understand the marginal effect of a single predictor on model prediction outcomes.

Unlike the factors of the built environment, factors of land cover and landscape index have a slighter and unclear impact on soundscape perception. To name just one sample for each, a higher proportion of forest up to 40 percent or a higher value of Shannon’s diversity index up to 1.0 in the AU are more likely to indirectly form a comfortable soundscape (see Figure A4). In terms of other reference factors, teenagers between 12 to 18 are the most likely group to make a positive evaluation, while the elderly older than 60 are most likely to recognize the soundscapes as negative ones. However, the temporal scale fails to demonstrate any obvious dissimilarity among different seasons and hours (see Figure A5).

(2) The joint effect of paired predictors on soundscape comfort evaluation

**Figure 4:**
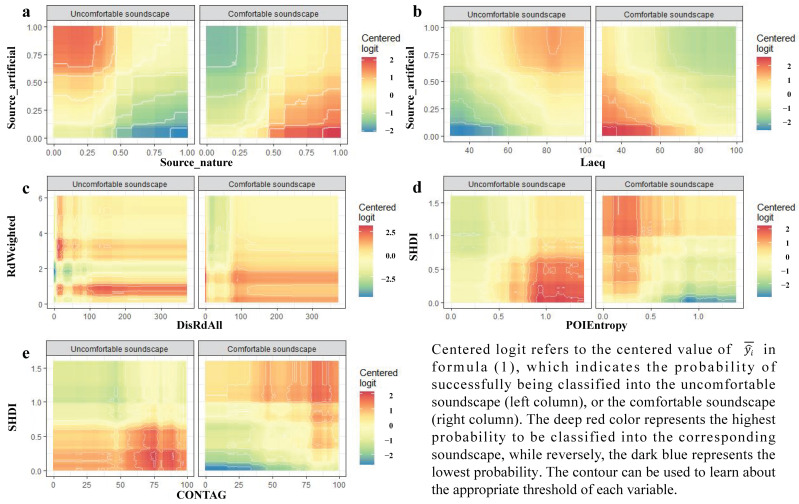
Partial dependence plot to understand the joint effect of paired predictors on model prediction outcomes.

Among the identified important variables, several paired predictors can be further selected to discuss the joint effects and the interaction mechanisms between them and the soundscape comfort evaluation. The mutual inhibition between sound sources is one of the interesting stories. Results show that in the condition of a low proportion of natural sounds (below 0.5) the probability of an uncomfortable soundscape will increase after the proportion of artificial sounds reaches 0.5, and vice versa: on the premise of a low proportion of artificial sound sources (less than 0.15), the soundscape tends to be more comfortable when the proportion of natural sound sources are closer to 1 (see Figure 4a). As for sound intensity and sound sources, the L_Aeq_ level higher than 65 dB and the proportion of artificial sound sources larger than 0.6 lead to the maximum probability of uncomfortable soundscape, while the L_Aeq_ level lower than 60 dB with the proportion of artificial sound sources lesser than 0.15 lead to the maximum probability of comfortable soundscape inversely (see Figure 4b).

The appropriate range of weighted road density in each AU is 1 to 2.5 km/km^2^, which is most unlikely to cause an uncomfortable soundscape. At the same time, its value below 2 km/km^2^ with a farther distance to the nearest road up to 100 m is preferred to create a comfortable soundscape (see Figure 4c).

The interaction between factors of the built environment and factors of landscape index can uncover the mutual influence of landscape substrate characteristics and its above human social-economic activities on soundscape perception. For instance, a low Shannon’s diversity index below 0.75 accompanied by a higher entropy of POI in each AU up to 0.75 can essentially result in an uncomfortable soundscape, while a low entropy of POI below 0.3 on the base of a high Shannon’s diversity index up to 1.0 can form the comfortable soundscape, indicating that urban planning should maintain a limited and well-organized land use structure with diverse land cover patches (see Figure 4d).

The interaction within landscape indexes can also produce clues about how the different dimensions of landscape substrate characteristics indirectly act upon soundscape perception. The plot shows that even with a high contagion up to 60 in each AU, a low Shannon’s diversity index below 0.75 may lead to an uncomfortable soundscape, while only in the case of both high contagion (up to 75) and high Shannon’s diversity (up to 1.0) the landscape context for a comfortable soundscape tends to be created (see Figure 4e).

## 4. Discussion

The conceptional implementations of Participatory Sensing through smartphone application programs can maximize the randomness of questionnaire inquiry and the diversity of the sample data. In our study, the PSS system recording sites scatter different downtown or suburban locations, providing various contexts for the soundscape which guarantees the precondition of the research engaging in unraveling the relationship between soundscape quality and diversified explanatory variables. We used a realistic case study of soundscape perception data in the Pearl River Delta of China to analyze its latent influence factors and mechanisms by organizing a large set of variables with 36 features. Following our model constructing process, we built an effective random forest model to predict the classification of soundscape perception quality, taking comfort as the proxy. By providing the order of variable importance for the overall soundscape comfort and a separate sequence set for each of the three comfort levels, we can observe how the 36 explanatory variables perform differently among these four perspectives of soundscape comfort. Furthermore, to enhance the feasibility of the random forest model when extensively applied in predicting the soundscape quality of new sites, we reorganized an optimal variable set with the 24 most important variables. Finally, through the implementation of partial dependence analysis, we explored the marginal effect of several single predictors and the joint effect of couples of paired predictors on model prediction outcomes, which can provide a deep insight into how the soundscape comfort responds to the subset of the features.

Compared to the previous work mentioned in the introduction section, we conducted a larger temporal–spatial scale of the study which can provide a diversified context with heterogeneous elements concerning people, activity, and place in space and time. Moreover, we have established an explanatory variable set with more abundant information extensively containing geospatial factors (including built environment elements, land cover elements, and landscape index), acoustic factors, demographic factors, and temporal factors, which can better depict a complete description of how the variables make up the certain context, as well as how they influence sounds and the acoustic environment through the context. Furthermore, the response we are concerned about is the soundscape comfort in comparison with the work by Mennitt et al., taking sound pressure level as the response [39]. We directly focus on the perception of soundscape, which can better obtain the perception or experience or understanding of soundscape from a humanistic perspective. In general, the constructed model strives for an approach to successfully predict the soundscape quality based on the optimized data set of 24 explanatory variables. Urban planners can utilize this model to evaluate the citizens’ soundscape perception in advance, and adjust the urban planning at the very beginning of the planning stage. Urban managements can also rapidly assess the citizens’ soundscape evaluation simply based on the 24 variables which can be regarded as the supplementary reference of the field questionnaires.

Some limitations in the current study should be highlighted. Though we have articulated in which underlying way the explanatory variables play their roles in the soundscape, we still found some difficulties in explaining the response for a few variables, especially for the two less important factors—landscape index and types of land cover. We speculated that this is because of the insufficient background information within the inquiry, and thus, the subjects’ perception and answers towards comfort may largely be affected by the instantaneous sound events or even their temporal emotions rather than the macroscopical and static features of landscape and land cover.

Given the aforementioned shortcomings and challenges, we also explored several directions for future work. In the aspects of participatory sensing, the questionnaire inquiry can be designed to focus on more dimensions of response in addition to soundscape comfort. In addition, we can construct the user’s profile as the complement variable sets containing socio-cultural factors through cluster analysis, which can help to prove the importance of socio-cultural factors in the interpretation of the acoustic environment [45]. Above all, we can provide more background information in the inquiry and guide the subjects to consider the imperative but overlooked surrounding context, which is essential for exploring the influence of geospatial factors on soundscape quality. As for the aspects of selecting explanatory variables, to improve the interpretability, it is necessary to exploit more data sources to ensure the effectiveness of the variable measurements and make them more intuitive and convenient when applied in the soundscape planning practice. Through these improvements, we believe that our model will have greater potential and provide more valuable references for urban planning and landscape management from the perspective of auditory and soundscape perception quality.

## 5. Conclusions

Sound is a fundamental aspect of nature and can be drastically affected by a variety of human activities. Thus, setting the soundscape and its hidden influence factors together in the framework of coupled natural–human systems can help to clarify what constitutes a high-quality soundscape in human habitats. The random forest model has confirmed its validity as a classification tool for soundscape quality (F1 score = 0.61) and has proven its ability to handle a large set of various explanatory variables and randomly collected sample data. Despite the failure to perform a significantly high prediction accuracy, overall, our methodology is sound and appears to have a heuristic value for similar research by providing an empirical case study.

Results of random forest model show that the acoustic factors are the most important factors for successfully classifying soundscape comfort, subsequently ranking by built environment elements, temporal factors, and demographic factors; landscape index and land cover type seem to have unclear importance. In addition, the results of the influence mechanisms point out several referable thresholds which can indicate the appropriate category, value, or range of those geospatial and acoustic factors to maintain a perceived comfortable and high-quality soundscape. The valuable references and refined keypoints for soundscape management and urban planning can be concluded as follows:

- more natural sound sources (>60%);
- a moderate proportion of human sound (20–60%);
- fewer artificial sound sources with as low sound level as possible;
- combination of more natural sounds (>60%) with fewer artificial sounds (<15%) or less artificial sounds (<15%) based on lower sound level (<60 dB);
- a lower road density (<15 km/km^2^) or a lower weighted road density (<2 km/km^2^);
- combination of a lower weighted road density (<2 km/km^2^) with a farther distance of human activity spaces from the nearest roads (>100 m);
- a limited POI entropy (<0.4);
- more green spaces, scenic spots, and accessible public administration and service spaces rather than industrial or transportation areas;
- combination of a higher Shannon’s diversity index (>1.0) with lower POI entropy (<0.3);
- combination of a high Shannon’s diversity concerning heterogeneous patches and a high contagion among homogeneous patches.

Our success demonstrates that the random forest model soundscape perception quality is well poised to integrate with diversified variables like geospatial factors, acoustic factors, demographic factors, and temporal factors. It can be utilized to act as an innovative indicator metric and pragmatic tool, and assist landscape and urban planners to build a more livable human habitat based on a humanistic consideration for human environment perception. In the future, we plan to extend the research to the worldwide range, as well as making the comparison among study areas with different characteristics, and finally propose the specific environmental planning and management of public health related to soundscape quality for the different types of human habitats.

## Figures and Tables

**Table 1:** Potential explanatory variables.

Variable Type	Variable	Description or Evaluation Indices	Units
Geospatial factors	Built environment		
	RdAll	Road density, sum of road lengths (all roads) divided by AU.	km/km²
	RdWeighted	$$\mathrm{RdWeighted}=\frac{L_{1}\times W_{1}+L_{2}\times W_{2}+\ldots +L_{n}\times W_{n}}{A},$$ where RdWeighted means the road density of each AU, A is the total area of AU, $L_{n}$ refers to the length of each road within per AU, and $W_{n}$ is the weight coefficients of the corresponding road hierarchy	km/km²
	RdMajor	Road density, sum of road lengths (major roads only) divided by AU	km/km²
	RdExpress	Road density, sum of road lengths (express roads only) divided by AU	km/km²
	DisRdAll	Distance to the nearest road (all roads)	m
	DisRdMajor	Distance to the nearest road (major roads only)	m
	DisRdExpress	Distance to the nearest road (express roads only)	m
	BldStructure	GB=∑i=1n(HnSn)n, where $G_{B}$ means the average three-dimensional building structure indices of each AU, $H_{n}$ and $S_{n}$ are the floor number and area of each building, n is the number of buildings in each AU. A higher value of BldStructure may cause larger acoustic energy due to the stronger reverberation between buildings with more floors and the less resistance of buildings with a smaller floor area	floor/km²
	POIEntropy	$$\mathrm{POIEntropy}=-\sum_{i=1}^{n}P_{i}\ln\left(P_{i}\right),$$ where POIEntropy means the functional entropy of POI in each AU, $P_{i}$ refers to the percentage of number for type i of POI within each AU, and n is the total number of POI types in each AU	/
	NLValue	nighttime light value	Nw cm^−2^ sr^−1^
	Landuse_site	The land use type of the recording site (1: residential; 2: business; 3: industrial; 4: public administration and service; 5: transportation; 6: green space and scenic spot; 8: farmland and wasteland)	/
	Landuse_context	The overall land use type in the AU, divided into three main categories: single functional area with certain land use type dominating, double functional area with two dominating land use type, and mixed functional area with comprehensive land use (1: residential; 2: business; 3: industrial; 4: public administration and service; 5: transportation; 6: green space or scenic spot; 7: mixed functional area; 8: farmland or wasteland; 9: residential–industrial; 10: residential–public administration and service; 11: residential–transportation; 12: residential–green space and scenic spot; 13: business–industrial; 14: business–public administration and service; 15: business–transportation; 16: business–green space and scenic spot; 17: industrial–public administration and service; 18: industrial–transportation; 19: industrial–green space and scenic spot; 20: business–farmland and wasteland; 21: public administration and service-green space and scenic spot; 22: transportation–farmland and wasteland; 23: residential–business)	/
	Urban location	The location of the recording site (1: downtown; 0: suburban).	/
	Land cover		
	LUCC_site	The land cover type of the survey site (10: cultivated land; 20: forest; 30: grassland; 40: shrub land; 60: water bodies; 80: artificial surface)	/
	Artificial surface	Proportion of artificial surface	%
	Cultivated land	Proportion of cultivated land	%
	Grassland	Proportion of grassland	%
	Forest	Proportion of forest	%
	Shrub land	Proportion of shrub land	%
	Water bodies	Proportion of water bodies	%
	Landscape Index *		
	PD	Patch density, $\mathrm{PD}=\frac{N}{A}$	n/100 ha
	ED	Edge density, $\mathrm{ED}=\frac{E}{A}10^{6}$	m/ha
	AREA_MN	Mean patch size, $\mathrm{MPS}=\frac{A}{N}10^{-6}$	ha
	LPI	Largest patch index, $\mathrm{LPI}=\frac{\max{\left(a_{\mathrm{ij}}\right)}_{j=1}^{n}}{A}\left(100\right)$	%
	CONTAG	Degree of contagion of land cover, $\mathrm{CONTAG}=\left[1+\overset{m}{\underset{i=1}{\sum }}\overset{n}{\underset{j=1}{\sum }}\frac{P_{\mathrm{ij}}\ln\left(P_{\mathrm{ij}}\right)}{2\ln\left(m\right)}\right]$ Pij=PiPji Pji=mijmi	/
	LSI	Landscape shape index, $\mathrm{LSI}=\frac{0.25E}{\sqrt{A}}$	/
	SHDI	Shannon’s diversity index, $\mathrm{SHDI}=-\overset{m}{\underset{i=1}{\sum }}\left[p_{i}\ln\left(p_{i}\right)\right]$	/
	PR	Patch richness, PR=m	/
Acoustic factors	Source_nature	Percentage of natural sound sources based on subjective evaluation (including wind; water; rain, insects, animal, and birds)	%
	Source_human	Percentage of sound sources from humans based on subjective evaluation (including speech, playing, and footstep)	%
	Source_artificial	Percentage of sound sources from artificial events based on subjective evaluation (including traffic, construction, music, machine, and airplane)	%
	L_Aeq_	SPL calculation: A-weighted equivalent continuous sound level	dB
Demographic factors	Age	Age group (1: younger than 12; 2: age between 12–18; 3: age between 19 and 20; 4: age between 31 and 40; 5: age between 41 and 50; 6: age between 51 and 60; 7: older than 60)	/
	Gender	Gender (1: men, 0: women)	/
Temporal factors	Diurnal	Dawn (1: 4 a.m. to 8 a.m.), diurnal (2: 8 a.m. to 4 p.m.), dusk (3: 4 p.m. to 8 p.m.), or nocturnal (4: 8 p.m. to 4 a.m.)	/
	Seasonal	Spring (1), summer (2), autumn (3), or winter (4)	/

* the detailed descriptions can be referenced in https://doi.org/10.2737/PNW-GTR-351, accessed on 10 August 2022.

**Table 2:** A subset of the explanatory variables for the three levels of comfort score ranked by relative importance. The most important variable is in the first line and subordinate ones are subsequently following in the decreasing order.

	Uncomfortable	Moderate	Comfortable
1	Source_artificial **	Age **	Source_nature **
2	L_Aeq_ **	BldStructure **	Source_artificial **
3	Source_nature **	Source_human **	L_Aeq_ **
4	Source_human **	Landuse_context **	Landuse_context **
5	RdAll **	DisRdMajor **	DisRdExpress **
6	NLValue*	Cultivated land **	NLValue **
7	AREA_MN **	L_Aeq_ **	RdWeighted **
8	Cultivated land **	NLValue **	Landuse_site *
9	RdWeighted **	RdMajor **	RdAll **
10	Age **	POIEntropy **	Seasonal **
11	POIEntropy **	Shrub land **	PD **
12	DisRdExpress*	DisRdExpress **	AREA_MN **
13	CONTAG **	RdWeighted **	Forest **
14	Diurnal **	Landuse_site **	DisRdAll *
15	Landuse_context (ns)	Seasonal **	RdMajor **
16	PD **	Forest **	Cultivated land **
17	SHDI **	Artificial surfaces **	RdExpress *
18	RdExpress *	Source_artificial **	BldStructure *
19	DisRdAll (ns)	Diunal **	LPI **
20	Shrub land **	RdAll*	Artificial Surfaces **
21	Landuse_site *	DisRdAll *	CONTAG*
22	Seasonal *	LPI **	Grassland **
23	RdMajor (ns)	Urban location **	SHDI **
24	BldStructure (ns)	Gender **	DisRdMajor *
25	ED *	SHDI **	POIEntropy (ns)
26	Grassland (ns)	Grassland *	Age (ns)
27	Forest (ns)	ED **	Diunal (ns)
28	LSI (ns)	PD *	LSI *
29	DisRdMajor (ns)	CONTAG (ns)	ED (ns)
30	Artificial surfaces (ns)	LUCC_site *	Shrub land (ns)
31	Gender (ns)	PR. **	PR. (ns)
32	Water bodies (ns)	LSI *	Source_human (ns)
33	PR (ns)	Water bodies (ns)	Urban location (ns)
34	LPI (ns)	AREA_MN (ns)	Gender (ns)
35	LUCC_site (ns)	RdExpress (ns)	Water_bodies (ns)
36	Urban location	Source_nature (ns)	LUCC_type (ns)

**: *p*-value ≤ 0.01, *: *p*-value ≤ 0.05, ns: *p* > 0.05.

## Data Availability

The data sets generated during the current study are available from the corresponding author on reasonable request.

## Appendix A

In these groups of graphs, the x-axis represents the value or the categories of the variable annotated below, and the y-axis represents the probability to be classified into the uncomfortable soundscape (left column) or the comfortable soundscape (right column). The probability values are standardized to around zero, whereas we should not concern about the specific value, rather than focus on the tendency of the curve. The upward curve means the greater probability to be classified into the corresponding soundscape. The downward curve represents a decreasing tendency reversely. The hush marks at the bottom of each plot indicate the data distribution.
